# The effect of the injectable platelet-rich fibrin on peri-implant soft tissue phenotype: a preliminary, prospective, 12-month follow-up clinical study

**DOI:** 10.1007/s00784-026-06849-0

**Published:** 2026-03-30

**Authors:** Berceste Guler Ayyildiz, Seyma Eken, Busra Terzioglu

**Affiliations:** 1 Faculty of Dentistry, Department of Periodontology, Kutahya Health Sciences University, No: 65, Eskişehir Highway Boulevard, İnköy Neighborhood, Kutahya, Turkey; 2 Tavsanlı Vocational School of Health Services, Oral Health Department, Kutahya Health Sciences University, Kutahya, Turkey; 3 Oral and Dental Health Application and Research Center, Kutahya Health Sciences University, Kutahya, Turkey

**Keywords:** Minimal invasive procedure, Phenotype, Platelet-rich fibrin, Peri-implant, Pink esthetic score, Soft tissue

## Abstract

**Objective:**

The aim of this study was to assess the effectiveness of submucosal injectable platelet-rich fibrin(i-PRF) application on the peri-implant soft tissue phenotype and pink esthetic outcomes in thin and thick phenotypes.

**Materials and methods:**

The present study comprised 36 implants from 10 patients. Based on the probe visibility method, implants were classified into Group I (thin phenotype) or Group II (thick phenotype). i-PRF applications were performed three times at one-month intervals into the peri-implant keratinized submucosal and mucogingival junction region. Keratinized mucosal width (KMW), mucosal thickness (MT), and marginal soft tissue recession height (REC-H) and width (REC-W) were measured at baseline (T0), 1 (T1), 3(T3), 6 (T6), and 12 months (T12). Modified plaque index (mPI), modified gingival index (mGI), probing depth (PD), and pink esthetic score (PES) were recorded at T0 and T12. Statistical analyses were conducted utilizing Generalized Estimating Equations (GEE) in conjunction with Type III Wald Chi-square tests.

**Results:**

Intragroup evaluations revealed that PD ​​increased significantly between T0 and T12 in Group I (*p* = 0.02). Intra-group and inter-group post hoc analyses demonstrated that Group I exhibited statistically significant alterations in MT at all evaluated time points, with the exception of the interval between T6 and T12 (*p* < 0.05). In contrast, no statistically significant changes were observed in either MT or KTW in Group II (*p* > 0.05). Furthermore, significant intergroup differences in MT were identified at T0, T1, and T3 (*p* < 0.001, *p* < 0.001, and *p* = 0.005, respectively). Evaluation of the PES across all implants revealed statistically significant enhancements in the total PES, as well as in the soft-tissue colour and contour subcomponents (*p* = 0.00, *p* = 0.04, and *p* = 0.04, respectively).

**Conclusions:**

Multiple applications of peri-implant i-PRF may alter the mucosal phenotype, particularly in thin phenotype cases, and consequently may lead to changes in the pink aesthetic score. Further research with larger samples is required.

**Clinical relevance:**

Multiple i-PRF injections applied to peri-implant sites may modify the particularly thin phenotype and may offer a new approach for clinical applications in limited cases.

## Introduction

The peri-implant soft tissue phenotype (STP) consists of three main components: peri-implant keratinized mucosal width (KMW), mucosal thickness (MT), and supracrestal tissue height. The effect of modifying this structure on peri-implant tissue health is still debated in the current literature [1]. The literature reports that KMW < 2 mm is associated with an increased incidence of peri-implant diseases. In contrast, adequate buccal MT (≥ 2 mm) may help prevent esthetic complications and maintain long-term mucosal stability [2]. In implants with insufficient KMW, attached mucosa, and MT, soft tissue phenotype modification (STPM) is beneficial in supporting peri-implant health and improving patient comfort and hygiene procedures [3, 4].

Various surgical approaches have been investigated to date for the STPM [1, 5, 6]. While STPM therapy has gained increasing clinical attention, current approaches—primarily gold-standard surgical techniques such as autogenous soft tissue grafts, which are among the most effective methods for increasing KMW and MT—are invasive, require technical skill, extended surgical time, donor site morbidity, postoperative discomfort, the potential for esthetically undesirable scar tissue formation, such as a patchwork appearance, and are often associated with low patient compliance [1, 7–9]. Although these techniques are widely used, their clinical indications still remain uncertain [9]. A consensus report on peri-implant keratinized mucosa highlights new insights into wound healing and neovascularization, suggesting advances in developing effective soft tissue structures without the need for autogenous grafting [10].

One of the platelet concentrates, injectable platelet-rich fibrin (i-PRF), is a biological agent that can be applied in liquid form. The first preparation of i-PRF was described by Miron et al. [11] using low-speed and short-duration centrifugation (3 min at 700 rpm). i-PRF is an effective biostimulator that accelerates wound healing by supporting tissue regeneration and stimulating processes such as stem cell migration, collagen synthesis, cell proliferation, and angiogenesis, thanks to its high levels of white blood cells and growth factors [11, 12]. It also forms a tiny fibrin clot that functions like a dynamic gel and, most importantly, offers a simple, low-cost application regardless of the individual’s financial status [12]. To more clearly demonstrate its potential clinical benefits in regenerative dentistry, further studies examining the use of liquid blood concentrate formulations are required [11].

A novel, minimally invasive approach involving multiple i-PRF injections has been proposed to address the thin gingival phenotype around teeth [13]. Although numerous studies have established that multiple i-PRF injections increase gingival thickness (GT) and/or keratinized tissue width (KTW) by evaluating their effect on the gingival phenotype of natural teeth [14–24], there is a lack of evidence regarding their effectiveness in peri-implant tissues. Notably, the impact of i-PRF on pink esthetic outcomes has not been investigated [14–24]. Due to the distinctive histological attributes of peri-implant mucosa – particularly its scar-like structure, parallel alignment of supracrestal fibers, and diminished vascularization relative to the periodontal ligament-dependent blood supply of natural teeth—the regenerative response of peri-implant tissues to i-PRF may differ substantially [25, 26]. Furthermore, to our knowledge, although the literature documents peri-implant STPM using surgical interventions [1, 5, 6], studies evaluating it with minimally invasive treatments have not been encountered. This study aims to evaluate the effectiveness of multiple i-PRF injections administered around implants with both thin and thick peri-implant phenotypes on STPM and pink esthetic outcomes. Accordingly, this research seeks to address existing gaps in the literature by providing a comprehensive analysis of the clinical benefits associated with multiple i-PRF injections. The null hypothesis of this study is that multiple i-PRF injections have no significant difference in STP and pink esthetics outcomes between peri-implant thin and thick phenotypes.

## Materials and methods

### Study design

This study was designed as a prospective controlled clinical trial with a 12-month follow-up. This study was performed in line with the principles of the CONSORT statement and the revised principles of the Helsinki Declaration (2013). Approval was granted by the Clinical Research Ethics Committee of Kütahya Health Sciences University, Turkey (No: 2022-17/08). This study was registered with ClinicalTrials.gov (No. NCT06753396), and informed consent was obtained from all individual participants included in the study. 

### Participants and setting

This study was conducted between 2022 and 2025 at the Department of Periodontology, Faculty of Dentistry, Kütahya Health Sciences University. The phenotype of implants that met the eligibility criteria was initially determined using the “probe visibility” method [27, 28]. To ensure objective accuracy and reproducibility, this clinical assessment was cross-verified by measuring MT with direct transgingival probing using an endodontic spreader and a digital caliper, as detailed in the ‘Data Collection’ section. Based on the combined assessment, the phenotype of each implant was assigned to one of the following groups:

- Group I: Defined as MT ≤ 1 mm, confirmed by the visibility of the periodontal probe through the sulcular tissue.
- Group II: Defined as MT > 1 mm, where the periodontal probe was not visible through the tissue.

### Eligibility criteria

Patient-level inclusion criteria were defined as follows: (1) age > 18 years; (2) systemically healthy or had controlled systemic disease; (3) patients who were willing to cooperate with the requirements of the study protocol, and to provide an appropriate written informed consent; (4) non-smokers or smokers of less than 10 cigarettes per day; (5) no conditions that may cause blood clotting problems. Implant-level inclusion criteria were defined as follow: (1) functionally loaded Nobel Biocare implants for at least one year; (2) peri-implant MT less than 2 mm; and (3) the absence of both peri-implant mucositis and peri-implantitis (the absence of inflammation, no bleeding on probing, and no suppuration and probing depth (PD) < 3 mm) which were defined according to the 2017 World Workshop on the Classification of Periodontal and Peri-Implant Diseases and Conditions [3].

**Fig. 1:**
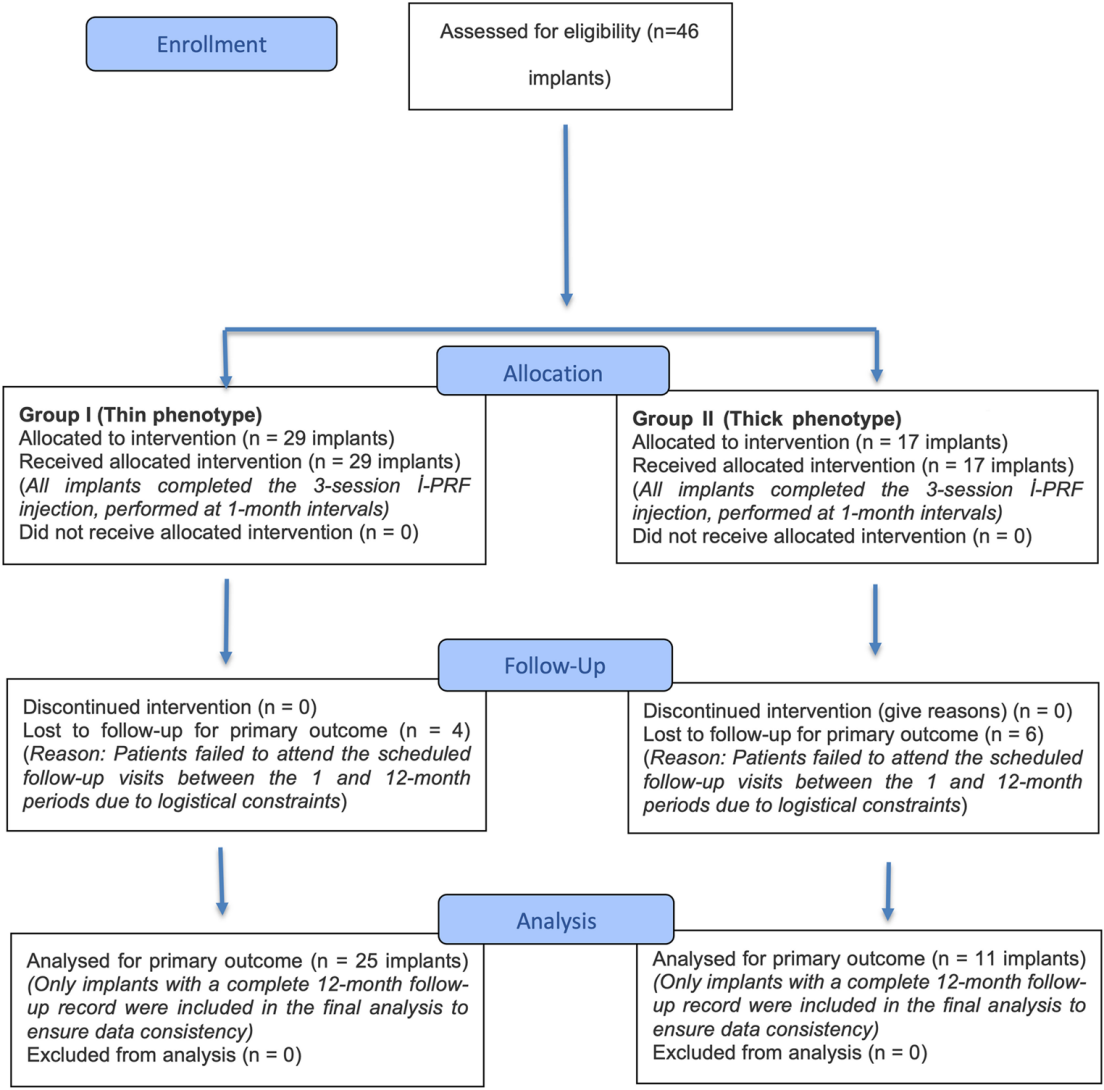
The study flowchart

**Fig. 2:**
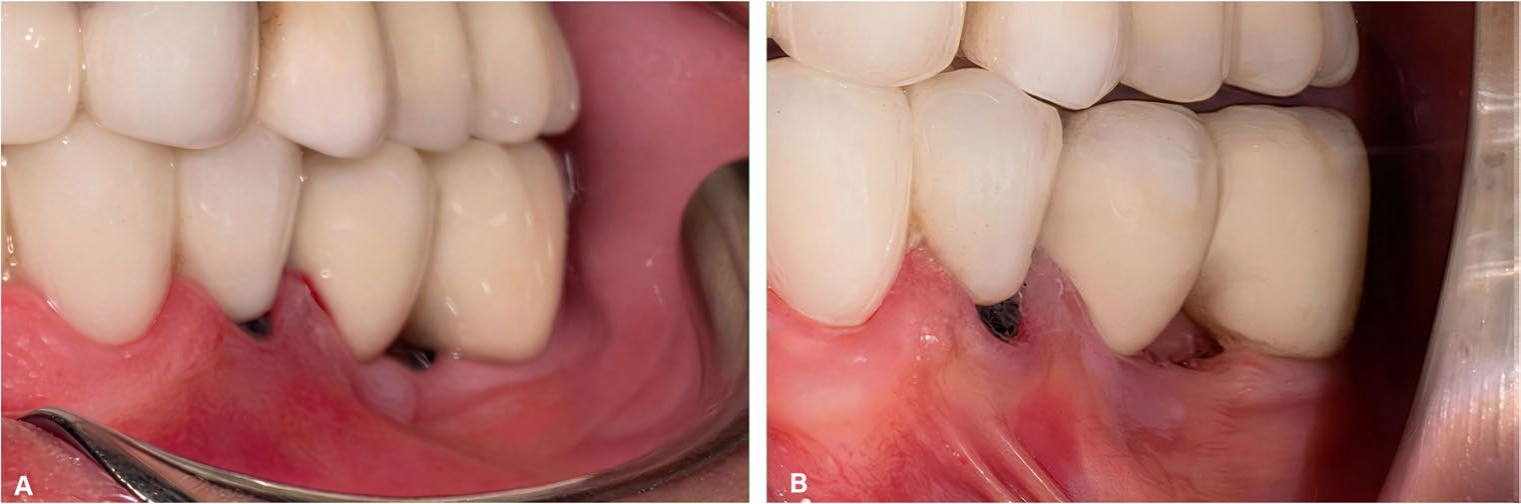
Pre-application (**A**) and post-application (**B**) views of the i-PRF procedure

**Fig. 3:**
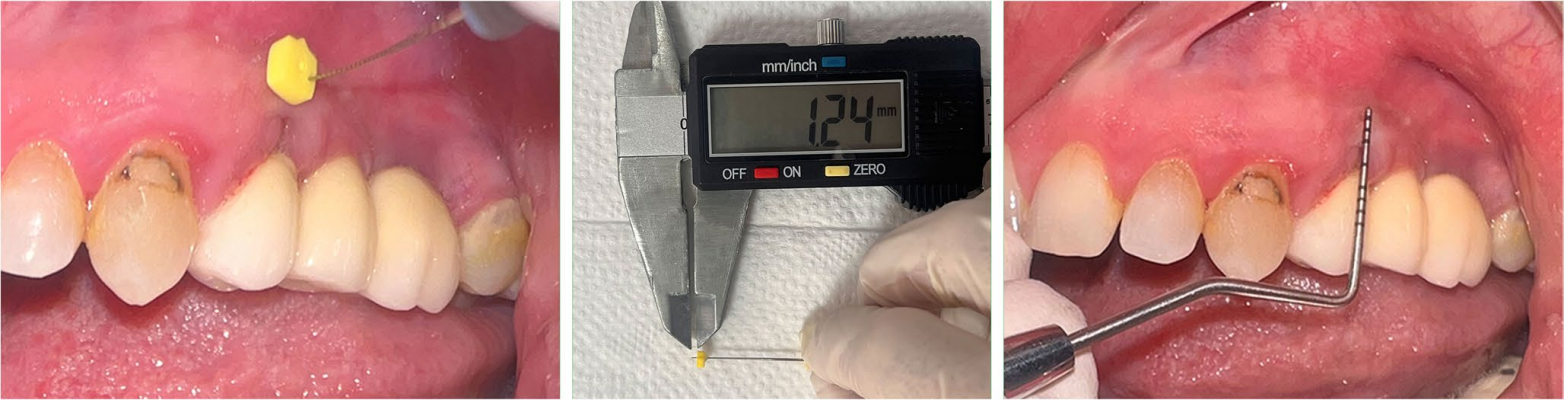
Mucosal thickness measured using a spreader and digital caliper; keratinized mucosa width assessed with a periodontal probe

Patient-level exclusion criteria were as follows: (1) heavy smoking (> 10 cigarettes/day); (2) uncontrolled systemic disease (e.g., diabetes mellitus with HbA1c ≥ 7.0%); (3) patients taking any systemic medications that could affect the healing of the peri-implant tissues (e.g., phenytoin, calcium channel blockers, cyclosporin, etc.); (4) platelet dysfunction syndrome or critical thrombocytopenia; (5) unavailability to attend follow-up appointments; (6) pregnant or lactating females; (6) immunocompromised (e.g., HIV) or under treatment with systematic corticosteroids and immunosuppressive drugs; (7) taking any medication that may cause gingival enlargement or blood thinners (NSAIDs including aspirin, and anticoagulants); (8) bruxism; (9) bisphosphonate treatment or prior radiation of the jaws. Implant-level exclusion criteria were as follow: (1) adequate peri-implant KMW and MT(> 2 mm), (2) implants positioned too buccally (subjective evaluation); (3) history of soft tissue augmentation at the site of interest; (4) severe trauma to the implant site; (5) the presence of an overhanging restoration preventing accurate probing; (6) implants that had undergone previous periodontal surgery (Fig. 1, 2, and 3).

### Preparation and application of i-PRF

A comprehensive medical history was obtained from all patients, and oral hygiene instructions were provided following Phase 1 periodontal treatment. The same procedures were applied in both groups four weeks after the completion of Phase 1 treatment.

**Fig. 4:**
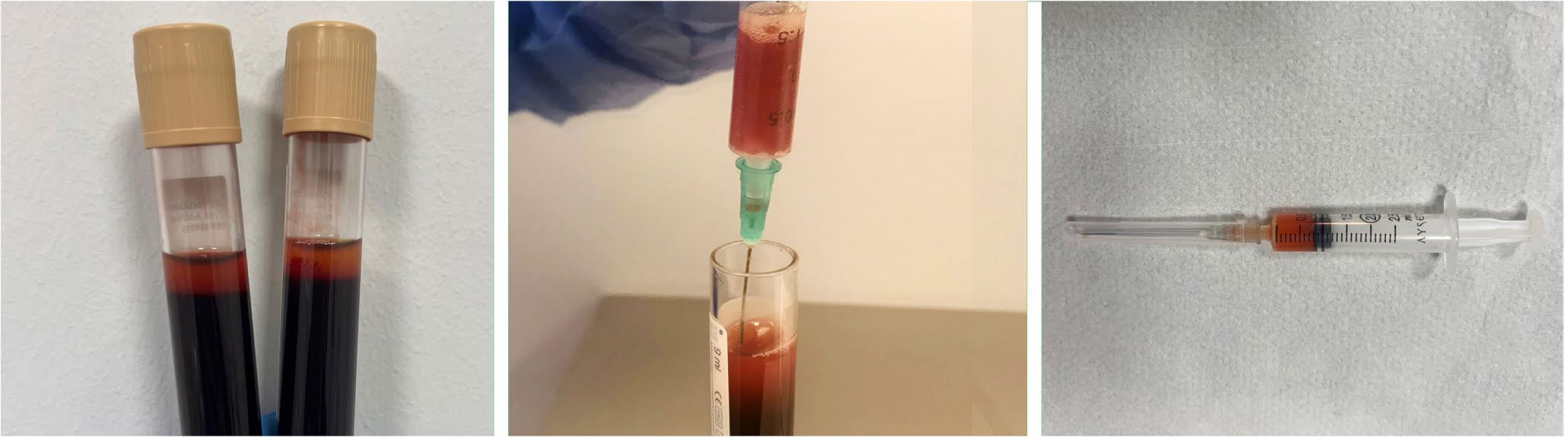
Obtaining i-PRF

**Fig. 5:**
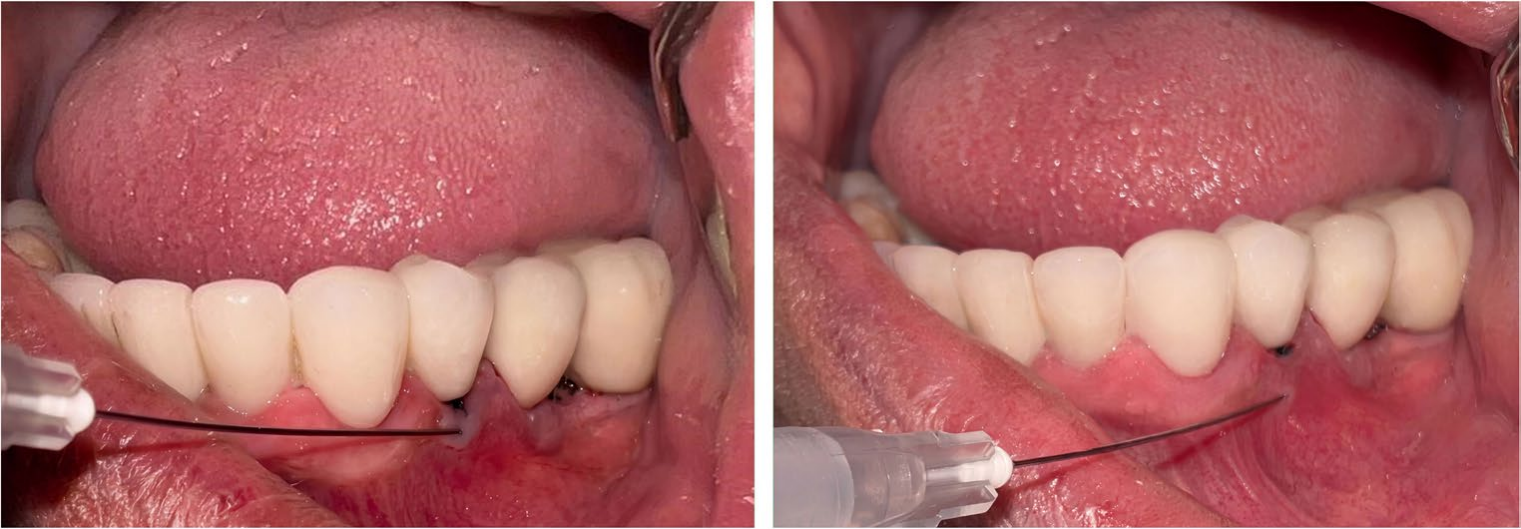
i-PRF injection into keratinized mucosa and mucogingival junction

The same periodontist (B.G.A.) conducted all procedures involving i-PRF employing a standardized protocol across all patients. Before the procedure, 10 mL of venous blood was drawn from each patient using a Vacusera^®^ blood collection system (Disera, Izmir, Turkey) with a 21-gauge butterfly needle and adapter. Samples were collected into 11 mL gamma-sterilized i-PRF tubes (BD Vacutainer^®^ I-Model, Becton Serum Blood Collection Tubes, Dickinson & Company, Franklin Lakes, NJ, USA) without anticoagulant at room temperature. To obtain i-PRF, the blood samples were processed in an NF 800R centrifuge device (Nüve, Ankara, Turkey) using a fixed-angle rotor. The centrifugation was performed at 700 rpm for 3 min (60 × g) at room temperature, following the protocol described by Miron et al. [11]. Following centrifugation, the upper orange/white translucent liquid phase (i-PRF), located above the red phase at the base, was carefully drawn into a 2.5-mL sterile dental syringe (Berika, Konya, Turkey) using 30-gauge needles (Fig. 4) and immediately applied to the site to ensure clinical efficacy. Infiltration local anesthesia was performed using articaine hydrochloride with epinephrine (Ultracaine DS Forte; Sanofi-Aventis, Frankfurt, Germany) at all implant sites. The prepared i-PRF was administered via submucosal injection in the peri-implant mid-vestibular/mid-buccal mucosa site, at the midpoint between the mucosal margin and the MGJ, and directly at the MGJ, using a 21-gauge needle. The injection was executed slowly and perpendicularly, with the needle being slightly withdrawn upon contact with hard tissue, until the blanching and fullness of the gingiva were observed. This procedure was repeated three times at one-month intervals (Fig. 5) [19].

### Data collection

Before the procedure (T0), the modified plaque index (mPI) [29], modified gingival index (mGI) [30], PD, vestibular sulcus depth (VSD), KMW, MT, marginal soft tissue recession height (REC-H), and marginal soft tissue recession width (REC-W) were measured from all implant sites. STP was evaluated using the “probe visibility” method as clinically [27, 28]. Following the procedure, KMW, MT, REC-H and REC-W parameters were assessed at 1 month (T1), 3 months (T3), 6 months (T6), and 12 months (T12). In addition, mPI, mGI, and PD were evaluated exclusively at T12. The pink esthetic score (PES) was assessed both clinically and from standard photographs obtained at T0 and T12 from the implant sites (Fig. 2) [31]. The implant location (maxilla or mandible) and implant position were recorded by region: anterior (incisor and canine positions) or posterior (premolar and molar positions). Clinical parameters were measured using a Williams-type probe (Nordent Manufacturing Inc., Elk Grove Village, Illinois, USA) with a diameter of 0.5 mm and 1 mm apart. Peri-implant MT was measured using an endodontic spreader (No. 15) and a digital caliper with 0.01 mm precision (Fig. 3).

- STP: The visibility between peri-implant tissues was measured using a color-coded periodontal probe and and then validated with transgingival probe method MT measurements. It was categorized as thin phenotype (Group I) and thick phenotype (Group II). Thin phenotype: Colour-coded periodontal probe visibility between implant tissues is high and MT ≤ 1 mm; Thick phenotype: Colour-coded periodontal probe visibility is low and MT > 1 mm [27, 28].
- KMW: The apico-coronal distance from the mucosal margin to the mucogingival junction (MGJ) at the mid-vestibular/mid-buccal region was measured in mm using a Williams-type probe.
- MT: In the mid-vestibular/mid-buccal region, a 3 mm diameter silicone-tipped endodontic spreader (No. 15) was placed vertically until a hard surface was felt, ensuring tight contact with the soft tissue surface, at the midpoint between the mucosal margin and the MGJ. The penetration depth between the silicone disc and the spreader tip was measured in mm using a digital caliper with a sensitivity of 0.01 mm [8].
- REC-H: Apico-coronal distance, defined as the distance to which the prosthetic abutment or implant neck is exposed, measured with the probe (in mm) [32].
- REC-W: Mesio-distal distance, defined as the distance to which the prosthetic abutment or implant neck is exposed, measured with the probe (in mm) [32].
- PES: Seven parameters related to the tissues surrounding the implant restoration, as defined by Fürhauser et al. [31] were evaluated. These parameters are: mesial papilla, distal papilla, soft-tissue level, soft-tissue contour, alveolar process deficiency, soft-tissue color, and texture. Each variable was measured using a 0-1-2 scoring system (0 = lowest, 2 = highest). The patient’s soft tissue color was assessed at T0 and T12, and all parameters were evaluated by calibrated investigators using standard photographs taken at T0 and T12.

### Primary and secondary outcomes

The primary outcome of this study is to evaluate the effect of multiple i-PRF injections applied to both thin- and thick-phenotype implants on MT at 12 months. Secondary outcomes include changes in KMW, clinical peri-implant parameters (mPI, mGI, PD), and PES parameters in both groups, as well as the potential impact of these changes on STP. These parameters were selected to provide a comprehensive assessment of the biological stability, clinical efficacy, and esthetic success of STP.

### Sample size

The primary outcome (MT) of the study was the basis upon which the differences between independent groups, time periods, and their interaction effects were examined. Accordingly, using the G-Power 3.1.9.2 program [33], at a 95% confidence level (α = 0.05), the standardized effect size (0.967) was calculated from a previously published, similar study [16]. The minimum sample size required to achieve 80% power was determined to be 18 for each time point and each group.

### Intra- and inter-examiner calibration

All clinical parameters were measured by two calibrated researchers (S.E. and B.T.). To ensure the reliability and reproducibility of the clinical measurements, both intra-examiner and inter-examiner calibration procedures were performed before the study. During the calibration phase, the examiners performed duplicate measurements of MT and KMW with a two-week interval in 10 patients with dental implants who were excluded from the main study. The intraclass correlation coefficient (ICC) values demonstrated excellent intra-examiner reliability, with ICCs of 0.979 (95% CI: 0.948–0.992) and 0.987 (95% CI: 0.968–0.996) for MT, and 0.991 (95% CI: 0.975–0.998) and 0.983 (95% CI: 0.955–0.994) for KMW. Inter-examiner reliability yielded ICC values of 0.958 (95% CI: 0.897–0.983) for MT and 0.971 (95% CI: 0.931–0.989) for KMW. For the subjective evaluation of PES, two examiners (S.E. and B.T.) underwent a preliminary calibration session using clinical photographs of five independent cases. During the study, any disagreements between the examiners were resolved through joint re-evaluation of the photographs until a consensus was reached for the final score.

### Statistical analyses

The study presented descriptive statistics of the data, including the numbers, percentages, means, standard deviations, medians, minimums, maximums, and 25% and 75% percentiles. The normality of the data distribution was assessed using the Shapiro-Wilk test. To account for the correlated nature of repeated soft tissue parameters, factors affecting clinical outcomes were analyzed using Generalized Estimating Equations (GEE) with a first-order autoregressive [AR(1)] working correlation matrix. The model included study groups, time points, and group x time interaction as predictors. The statistical significance of the main effects and interactions in the model was evaluated using Type III Wald Chi-Square tests (*p* < 0.05). Post hoc pairwise comparison tests were adjusted with the Bonferroni correction. In comparisons between two independent groups, the Independent Samples t-test was used when the normality assumption was met, and the Mann-Whitney U test was used when it was not. Additionally, for intra-group comparisons between two time points, the Wilcoxon signed-rank test was utilized. Relationships between variables were assessed using Pearson correlation for normally distributed data and Spearman correlation for data that did not meet the assumption of normality. All statistical analyses were conducted using SPSS Statistics version 27 (IBM Corp. Released 2020. IBM SPSS Statistics for Windows, Version 27.0. Armonk, NY: IBM Corp.), and a p-value < 0.05 was considered statistically significant.

## Results

### Demographic data and implant characteristics

Initially, 13 patients with 46 implants that met the selection criteria were enrolled in the study. During follow-up, 3 patients (10 implants) were excluded due to non-attendance and insufficient data. Therefore, the analysis was conducted on 10 patients with 36 implants in total (5 female and 5 male; aged 33–66 years; mean age = 49.22 ± 10.74 years), with 25 implants in Group I and 11 implants in Group II. This final sample size of 36 implants achieved the targeted 80% statistical power with a 5% margin of error. The lower number of implants in Group II compared to Group I resulted from random follow-up losses. To minimize the potential impact of this numerical imbalance on statistical outcomes, generalized estimating equations (GEE), which are robust to group size differences, were used for analysis. The study flow chart is presented in Fig. 1.

Table 1 shows demographic data and the distribution of implant characteristics.

**Table 1:** Demographic data and implant characteristic

	Group I (*N*=9, n=25)	Group II (*N*=7, n=11)	Total implants (*N*=10, n=36)
Sex, *N* (%)			
Female	5 (55.6%)	4 (57.1%)	5 (50%)
Male	4 (44.4%)	3 (42.9%)	5 (50%)
Smoking status, *N* (%)			
Non-smoker	8 (88.9%)	6 (85.7%)	9 (90%)
Smokers of ≤10 cigarettes per day	1 (11.1%)	1 (14.3%)	1 (10%)
Total implant number, *n* (%)			
Anterior implants, *n* (%)	14 (56%)	5 (45.4%)	19 (52.8%)
Posterior implants, *n* (%)	11 (44%)	6 (54.6%)	17 (47.2%)
Maxillary implants, *n* (%)	18 (72%)	4 (36.4%)	22 (61.1%)
Mandibular implants, *n* (%)	7 (28%)	7 (63.6%)	14 (38.9%)

*N* Number of patients; *n* Number of implant; % Percentage

### Longitudinal analysis of soft tissue parameters: generalized estimating equations model analysis

The distribution of soft tissue parameters across groups and time periods is given in Table 2.

**Table 2:** Distribution of soft tissue parameters across groups and time-points

		T0		T1		T3		T6		T12	
	Group	Mean ± SD	M (25–75%)	Mean ± SD	M (25–75%)	Mean ± SD	M (25–75%)	Mean ± SD	M (25–75%)	Mean ± SD	M (25–75%)
KMW	Group I	2.5 ± 1.99	2(1–4)	2.7 ± 1.93	2(1–5)	2.75 ± 1.98	2(1–5)	2.98 ± 1.87	3(1.5-5)	2.69 ± 1.47	2.5(1.75-4)
	Group II	2.14 ± 0.87	2(1.5-3)	2.23 ± 1.1	2(1.5-3)	2.36 ± 1.03	2(1.5-3)	2.73 ± 1.08	3(2–3)	2.77 ± 1.06	3(2–4)
MT	Group I	0.68 ± 0.37	0.5(0.5-1)	0.86 ± 0.35	1(0.5-1)	1.04 ± 0.49	1(0.75-1)	1.35 ± 0.56	1(1-1.75)	1.5 ± 0.61	1.33(1.2–1.5)
	Group II	1.18 ± 0.28	1(1-1.5)	1.25 ± 0.3	1.5(1-1.5)	1.48 ± 0.43	1.5(1–2)	1.4 ± 0.44	1.39(1–2)	1.52 ± 0.4	1.39(1.2-2)
REC-H	Group I	0.46 ± 0.66	0(0–1)	0.34 ± 0.57	0(0-0.5)	0.32 ± 0,56	0(0-0.5)	0.34 ± 0.57	0(0-0.5)	0.54 ± 0.78	0(0–1)
	Group II	0.32 ± 0.56	0(0–1)	0.23 ± 0.41	0(0-0.5)	0.23 ± 0.41	0(0-0.5)	0.23 ± 0.41	0(0-0.5)	0.27 ± 0.52	0(0-0.5)
REC-W	Group I	0.9 ± 1.32	0(0–2)	0.84 ± 1.34	0(0–2)	0.8 ± 1.32	0(0–2)	0.88 ± 1.45	0(0–2)	1.36 ± 1.68	0(0–3)
	Group II	0.73 ± 1.27	0(0–2)	0.73 ± 1.27	0(0–2)	0.55 ± 1.04	0(0–1)	0.73 ± 1.27	0(0–2)	0.82 ± 1.4	0(0–3)

*Group I* defined as MT ≤ 1 mm, confirmed by the visibility of the periodontal probe through the sulcular tissue; *Group II* defined as MT > 1 mm, where the periodontal probe was not visible through the tissue; *KMW* Peri-implant keratinized mucosa width; *MT* Mucosal thickness; *REC-H* Marginal soft tissue recession height; *REC-W* Marginal soft tissue recession width; *M* Median; % Percentage; *SD* Standard deviation

Based on the generalized estimating equation model, the effects of study groups, time points, and their interactions on soft tissue parameters are presented in Table 3. For parameter KDW, only the main effect of time was statistically significant (*p* < 0.001). Regarding parameter MT, significant main effects were observed for group (*p* = 0.010) and time (*p* < 0.001), as well as a significant group × time interaction effect (*p* = 0.006). For parameters REC-H and REC-W, the main effects of time and group, and the time x group pairwise interaction effect were found to be statistically insignificant (*p* > 0.05).

**Table 3:** GEE Model Analysis of soft tissue parameters across groups and time periods

	Source	X^2^	df	*p*
KDW	Intercept	132.781	1	< 0.001*
	Group	0.389	1	0.533
	Time	35.993	4	< 0.001*
	Group × Time Interaction	3.935	4	0.415
MT	Intercept	507.468	1	< 0.001*
	Group	6.664	1	0.010*
	Time	74.476	4	< 0.001*
	Group × Time Interaction	14.450	4	0.006*
REC-H	Intercept	15.022	1	< 0,001*
	Group	0.742	1	0.389
	Time	6.375	4	0.173
	Group × Time Interaction	1.909	4	0.753
REC-W	Intercept	14.690	1	< 0.001*
	Group	0.323	1	0.570
	Time	8.768	4	0.067
	Group × Time Interaction	3.555	4	0.470

*p < 0.05, df: Degrees of Freedom, Generalized Estimating Equations (GEE) [AR(1)] and Type III Wald Chi-Square Test, *KMW* Peri-implant keratinized mucosa width; *MT* Mucosal thickness; *REC-H* Marginal soft tissue recession height; *REC-W* Marginal soft tissue recession width

### Intra-group and Inter-group Comparisons

Given the significant group × time interaction for parameter MT, post-hoc pairwise comparisons with Bonferroni correction were performed to group and time differences (Table 4). Intra-group comparisons revealed that Group I exhibited significant changes across multiple time intervals (*p* < 0.05). Specifically, significant differences were found between T0-T1 (*p* = 0.012), T0-T3 (*p* < 0.001), T0-T6 (*p* < 0.001), and T0-T12 (*p* < 0.001); however, no statistically significant difference was found between T6 and T12 (*p* > 0.05). In contrast, Group II showed no significant changes across any time interval (*p* > 0.05).

**Table 4:** Post-hoc pairwise comparisons of MT with bonferroni correction

		I	J	Mean difference (I-J)	*p*
Group	Group I	T0	T1	-0.180	0.012*
			T3	-0.360	< 0.001*
			T6	-0.668	< 0.001*
			T12	-0.818	< 0.001*
		T1	T3	-0.180	0.031*
			T6	-0.488	< 0.001*
			T12	-0.638	< 0.001*
		T3	T6	-0.308	0.004*
			T12	-0.458	< 0.001*
		T6	T12	-0.150	0.974
	Group II	T0	T1	-0.068	1.000
			T3	-0.295	0.102
			T6	-0.222	0.481
			T12	-0.335	0.113
		T1	T3	-0.227	0.905
			T6	-0.154	1.000
			T12	-0.267	0.608
		T3	T6	0.074	1.000
			T12	-0.040	1.000
		T6	T12	-0.114	1.000
Time	T0	Group I	Group II	-0.502	< 0.001*
	T1	Group I	Group II	-0.390	< 0.001*
	T3	Group I	Group II	-0.437	0.005*
	T6	Group I	Group II	-0.056	0.741

*p < 0.05, Bonferroni Correction as a Post Hoc Analysis following GEE analysis

Inter-group comparisons at each time point indicated that Group I had significantly lower than Group II at T0 (Mean Difference = -0.502, *p* < 0.001), T1 (Mean Difference = -0.390, *p* < 0.001), and T3 (Mean Difference = -0.437, *p* = 0.005). However, the inter-group difference was not statistically significant at T6 (Mean Difference = -0.056, *p* = 0.741). (Table 4)

Regarding clinical parameters mPI, mGI, and PD, no statistically significant differences were observed between the two groups at either T0 or T12 (*p* > 0.05). Nevertheless, intra-group analyses indicated that, in Group I, a statistically significant increase was observed exclusively in PD from T0 to T12 (*p* = 0.02) (Table 5).

**Table 5:** Intergroup and intragroup comparison of clinical parameters

	Group	T0 (Mean±SD)	T12 (Mean±SD)	T0-T12 P**
mPI	Group I	0.85±0.89	0.79±0.62	0.6
	Group II	0.65±0.92	1.09±0.63	0.18
P*		0.57	0.21	
mGI	Group I	1.22±0.79	0.71±0.53	0.07
	Group II	1.09±0.86	1.0±0.57	0.77
P*		0.67	0.17	
PD	Group I	2.53±0.73	3.13±0.98	0.02
	Group II	2.65±0.89	3.22±0.68	0.11
P*		0.69	0.74	
VSD	Group I	6.87±2.56		
	Group II	8.43±1.51		

*P* < 0.05, *Independent Sample t-test; **Wilcoxon signed-rank test. Group I: Defined as MT ≤ 1 mm, confirmed by the visibility of the periodontal probe through the sulcular tissue; Group II: Defined as MT > 1 mm, where the periodontal probe was not visible through the tissue; *SD* Standard deviation; *mPI* Modified plaque index; *mGI* Modified gingival index; *PD* Pocket depth; *VSD* Vestibular sulcus depth

### Confounding factors associated with KMW and MT

No significant associations were revealed for KMW or MT at T12 and variables including gender, presence of systemic medical conditions, implant position, or implant location (*p* > 0.05) (Table 6).

**Table 6:** Comparison of demographic data and implant characteristics with KMW and MT at T12

			Mean±SD	M (25-75%)	Test statistics	*p*
KMW (T12)	Sex	Male	2.64±1.49	2(1.63-3.5)	-0.300**	0.766
		Female	2.78±.25	2.75(2-4)		
	Systemic medical conditions	Healty	2.88±1.39	2.5(2-4)	1.42**	0.16
		Controlled systemic disease	2.13±1.03	2(1.4-2.75)		
	Implant position	Anterior	3.09±1.27	3(2-4)	0.34**	0.74
		Posterior	2.29±1.33	2(1.5-3)		
	Jaw location	Maxillary	3.07±1.45	2.75(2-4)	1.84**	0.07
		Mandibular	2.16±0.96	2(1.5-3)		
MT (T12)	Sex	Female	1.56±0.59	1.5(1.18-2)	-0.79*	0.44
		Male	1.46±0.53	1.33(1.2-1.5)		
	Systemic medical conditions	Healty	1.56±0.61	1.45(1.15-2)	-1.04*	0.32
		Controlled systemic disease	1.32±0.24	1.2(1.2-1.5)		
	Implant position	Anterior	1.44±0.47	1.33(1.2-1.5)	-0.86*	0.39
		Posterior	1.58±0.64	1.5(1.2-2)		
	Jaw location	Maxillary	1.51±0.65	1.36(1.2-1.5)	-0.31*	0.76
		Mandibular	1.49±0.38	1.4(1.2-2)		

*P* < 0.05, **Independent Sample T test, *Mann-Whitney U test, *KMW* Peri-implant keratinized mucosa width; *MT* Mucosal thickness; *M* Median; *SD* Standard deviation

### Pink esthetic score

The PES parameters and, along with their comparative analysis between T0 and T12, are presented in Table 7. Statistically significant differences were identified between T0 and T12 in PES–soft tissue contour, PES–soft tissue colour, and the total PES score (*p* = 0.04, *p* = 0.04, and *p* = 0.00, respectively).

**Table 7:** Patients’ PES measurements, time comparisons, and correlations

Distributions					Correlations			
					KMW (T12)		MT (T12)	
	Time	Min.-Max.	Mean ± SD (Median)	P*** (T0-T12)	r	*p*	r	*p*
PES-Mesial papilla	T0	0–2	1.39 ± 0.64(1)	0.08***	-0.03	0.88*	-0.03	0.86*
	T12	0–2	1.47 ± 0.61(2)		0.08	0.64*	-0.15	0.37*
PES-Distal papilla	T0	0–2	1.11 ± 0.57(1)	1.00***	0.19	0.25*	0.04	0.83*
	T12	0–2	1.11 ± 0.57(1)		0.19	0.25*	0.04	0.83*
PES-Soft tissue level	T0	1–2	1.36 ± 0.49(1)	1.00***	0.30	0.07*	0.25	0.14*
	T12	1–2	1.36 ± 0.49(1)		0.30	0.07*	0.24	0.14*
PES-Soft tissue contour	T0	1–2	1.22 ± 0.42(1)	0.04**	0.08	0.64*	0.14	0.40*
	T12	1–2	1.33 ± 0.48(1)		0.27	0.11*	0.27	0.11*
PES-Alveolar process deficiency	T0	1–2	1.53 ± 0.51(2)	1.00***	0.36	0.03**	-0.14	0.40*
	T12	1–2	1.53 ± 0.51(2)		0.36	0.03**	-0.14	0.40*
PES-Soft-tissue colour	T0	1–2	1.81 ± 0.40(2)	0.04**	-0.11	0.52*	0.01	0.97*
	T12	1–2	1.92 ± 0.28(2)		-0.15	0.36*	-0.13	0.44*
PES-Soft-tissue texture	T0	1–2	1.81 ± 0.40(2)	0.15***	-0.04	0.81*	0.02	0.90*
	T12	1–2	1.86 ± 0.35(2)		-0.17	0.30*	-0.17	0.31*
Total PES	T0	7–14	10.31 ± 1.80(10)	0.00**	0.27	0.11†	0.06	0.71†
	T12	7–14	10.58 ± 1.83(11)		0.27	0.10†	0.02	0.89†

**p < 0.05 †Pearson and *Spearman correlation, ***Wilcoxon signed-rank test, *PES* Pink esthetic score; *KMW* Peri-implant keratinized mucosa width; *MT* Mucosal thickness

### Correlation analysis

A moderate, statistically significant positive correlation was found between KMW at T12 and PES-Alveolar process deficiency at both T0 and T12 (*r* = 0.36, *p* = 0.03; Table 7).

No statistically significant relationships were found between KMW or MT measurements at T12 and clinical or soft tissue parameters (*p* > 0.05) (Table 8). Additionally, no significant relationships were observed between T12-T0 change in soft tissue parameters and Total-PES measurements (*p* > 0.05) (Table 9).

**Table 8:** Correlations between KMW and MT at T12 and clinical and soft tissue parameters

		KMW (T12)	MT (T12)
mPI	r	-0.149*	-0.038*
	p	0.387	0.827
mGI	r	0.036*	0.033*
	p	0.834	0.849
PD	r	0.110†	0.215†
	p	0.523	0.209
VSD (T0)	r	0.185*	0.131*
	p	0.410	0.561
REC-H (T0)	r	0.252*	0.044*
	p	0.139	0.797
REC-W (T0)	r	0.163*	0.059*
	p	0.343	0.734

*P* < 0.05, †Pearson and *Spearman correlation, *mPI* Modified plaque index; *mGI* Modified gingival index; *PD* Pocket depth; *VSD* Vestibular sulcus depth; *KMW* Peri-implant keratinized mucosa width; *MT* Mucosal thickness; *REC-H* Marginal soft tissue recession height; *REC-W* Marginal soft tissue recession width

**Table 9:** Correlations between total-PES and soft tissue parameters for T12-T0

T12-T0		Total-PES
KMW	r	0.13
	p	0.45
MT	r	-0.12
	p	0.48
REC-H	r	0.21
	p	0.20
REC-W	r	0.28
	p	0.09

*p < 0.05, Spearman correlation; *PES* Pink esthetic score; *KMW* Peri-implant keratinized mucosa width; *MT* Mucosal thickness; *REC-H* Marginal soft tissue recession height; *REC-W* Marginal soft tissue recession width

## Discussion

The present study evaluated the effects of submucosal multiple i-PRF injections, a minimally invasive technique, on STP and pink esthetic outcomes in peri-implant thin and thick phenotypes. Preliminary results indicate that the group-time effect is significant for the MT parameter. Post-hoc comparisons confirmed that, in the thin phenotype, MT value increase was statistically significant in all follow-up periods except the T6-T12 interval, however, MT values tended to stabilize at the end of follow-up. Analysis of all implants demonstrated that changes in soft tissue contour, color, and overall PES score were statistically significant from baseline to one year. The null hypothesis was partially rejected: multiple i-PRF injections did not significantly enhance overall clinical parameters in either phenotype, but improved pink esthetic outcomes in all implants and positively affected STP in the thin phenotype.

The Best Evidence Consensus developed in 2016, outlined strategies for maintaining periodontal and peri-implant health and introduced STPM therapy as a method to support gingival health [34, 35]. Recent developments in STPM therapy include non-surgical minimally invasive techniques such as microneedling (MN) [13], hyaluronic acid [14], and autologous platelet concentrates [13, 22, 23]. i-PRF, a liquid form of autologous platelet concentrate without anticoagulants or a fibrin matrix, is a promising tool in STPM therapy protocols due to its high concentration of regenerative cells and bioactive growth factors that support collagen production and tissue regeneration [11, 15, 20].

Özsağır et al. [13] and Fotani et al. [23] reported that multiple i-PRF injections in the thin gingiva phenotype led to an increase in GT and KTW in natural teeth. Current evidence indicates that i-PRF as an effective, minimally invasive approach for modifying thin periodontal phenotypes in natural teeth, enhancing soft tissue stability and reducing surgical interventions [11, 17, 21]. In many studies, i-PRF has been used alone or in combination with other methods [36]. A recent systematic review reported that i-PRF may offer superior esthetic outcomes compared to free gingival grafts [37]. In this respect, the present study represents the initial investigation of the effects of multiple i-PRF injections—a non-surgical, minimally invasive method—on peri-implant soft tissues.

Injectable-platelet rich fibrin is estimated to release growth factors over approximately 7 to 11 days. Therefore, i-PRF injections have been administered at 7-day intervals [20, 21, 23], in some studies, and at 10-day intervals in others [13, 17, 18]. In our study, i-PRF injections were administered around the implant at one-month intervals (three times) to reinforce tissue during periods of reduced growth factor levels. The mechanism of GT increase with i-PRF can be explained by the release of growth factors that trigger fibroblast proliferation over 5 days to 8 weeks, and the synthesis of collagen and elastin fibers as a result [19]. Additionally, fibroblasts promote neocollagenesis and neoangiogenesis, facilitating tissue remodeling from eight weeks to one year [38]. Soft tissue healing after peri-implant surgery comprises initial inflammation (first week), proliferation and neoangiogenesis (1–4 weeks), early remodeling (1–3 months), and maturation with neocollagenesis (up to 1 year), resulting in biological stability [39]. Furthermore, studies evaluating the effects of multiple i-PRF injections on the gingival phenotype of natural teeth have conducted follow-up evaluations at 1, 3, and 6 months [14–24]. To assess long-term effect of i-PRF on soft tissue stability, i-PRF application protocol were set as three times at one-month intervals and, follow-ups were scheduled at 1, 3, 6, and 12 months over one year.

One study reported a statistically significant reduction in both PI and GI after 3 months compared to baseline values [23]. Özsagir et al. [13] reported significant improvements in PI, PD, Bleeding on Probing (BoP), and clinical attachment level at 6 months. In contrast, two studies documented reductions in PI and GI during a 3-month follow-up period that did not reach statistical significance [20, 21]. Similarly, Faour et al. [14] in a comparative analysis of i-PRF and hyaluronic acid (HA), identified no significant intergroup differences in GI and PD. To our knowledge, there was no studies have examined the effect of VSD on i-PRF injections around natural teeth [14–24]. VSD is crucial for tissue stability; Halperin-Sternfeld et al. [40] reported that an insufficient peri-implant VSD increases the risk of mucosal recession and worsens BoP and GI. A consensus report indicated that thin phenotype implants also had less favorable clinical outcomes, including increased PD, BoP, inflammation, and mucosal recession [41]. In the present study, with limited data, an increase in PD was observed one year after i-PRF injection in the thin phenotype, suggesting that i-PRF injection may be inadequate for sustained soft-tissue stability. Additionally, no significant associations were identified between baseline VSD and other clinical parameters, KMW, or MT at 12 months. Discrepancies with the literature may reflect biological differences between implants and teeth, protocol heterogeneity, follow-up duration, and clinical variability.

Several studies conducted on natural teeth with thin phenotypes have reported a statistically significant increase in GT at all evaluation time points following multiple i-PRF injections [13–15, 18–20, 22, 24]. Yadav et al. [24] observed an increase from 0.77 mm to 0.93 mm at 6 months (20.77%), Adhikary et al. [15] from 0.55 mm to 0.69 mm at 6 months (25.45%), Soundarajan [18] from 0.46 mm to 0.62 mm at 3 months (34.78%), Özsağır et al. [13] a 44% increase at 3 months (stable at 6 months), and Tiwari et al. [20] from 0.65 mm to 1 mm at 3 months (53.84%). A systematic review and meta-analysis reported GT gains of 0.12 mm and 0.17 mm at 3 and 6 months, respectively, in studies with 6-month follow-up, whereas studies with 3-month follow-up showed gains of 0.38 mm and 0.26 mm at 1 and 3 months, respectively [36]. One study reported a 26.56% increase in GT at 3 months and a 29% increase at 6 months compared with baseline [19]. Among the studies reporting higher GT gains, Fotani et al. [23] reported an increase from 0.55 mm to 1.03 mm (87.27%) in 3 months, while Akolu et al. [21] an increase from 0.66 mm to 1.26 mm (90.9%) within 3 months. Furthermore, Shaker et al. [22] reported that i-PRF increased maxillary jaw GT from 0.71 mm to 0.85 mm (19.7%) and mandibular jaw GT from 0.5 mm to 0.8 mm (60%) at 3 months, and concentrated–PRF demonstrated greater efficacy than i-PRF. Furthermore, evidence indicates that adjunctive use of i-PRF with MN [15] or semi-surgical techniques [42] may enhance clinical outcomes. Current evidence demonstrates that the degree of GT increase varies according to the i-PRF application technique, the frequency of administration, and the duration of follow-up. In our study, a consistent increase in MT was observed around thin phenotype implants at all time evaluation points (except 6 months to 12 months), while MT remained stable in the last 6 months; no statistically significant increases were observed in thick phenotypes. Moreover, statistically significant differences were identified between the two groups at T0, T1, and T3, whereas no significant distinction was observed at T6. Notably, at T6, the values observed in the thin phenotype group reached levels comparable to those in the thick phenotype group. The thin phenotype increased from 0.68 mm to 1.5 mm at 12 months, with a rate of over 100%, while the thick phenotype group increased from 1.18 mm to 1.52 mm (28.81%). Relative to studies on thin-phenotype teeth, the greater increase in MT observed in thin-phenotype implants in our study likely reflects inherent histological and biomechanical differences between implants and natural teeth [43]. Specifically, the peri-implant connective tissue exhibits higher collagen content, altered vascularity due to the absence of the periodontal ligament (vascular drainage), and parallel organization of supra-crestal fibers (absence of injection resistance) [25, 26]. Collectively, these features may contribute to a more substantial adaptive response and greater tissue augmentation following treatment compared to natural teeth.

Some studies define the thick phenotype as ≥ 2 mm [4, 44, 45], while others define it as > 1 mm [27, 28, 46]. To our knowledge, the literature has shown that different threshold values ​​are used in the classification of STP, and there are also differences in the phenotype determination methods [27, 28, 44–47]. The 2017 World Classification Workshop recommends using ≤ 1 mm as the thin phenotype and > 1 mm as the thick phenotype in clinical practice [46]. In our study, a threshold value of 1 mm and the probe visibility method were used for STP assessment because it is more clinically and methodologically consistent. Accordingly, implants were classified into thin and thick phenotype groups. All implants had an MT of less than 2 mm. At the 12-month follow-up, 22.22% of implants reached an MT of ≥ 2 mm. Within thick phenotype (MT > 1 mm), the distribution of measurements showed that 45.45% of the sites (*n* = 5) were closely clustered near the 1.0 mm threshold. At 12 months, 63.63% of implants in the thick phenotype group achieved a MT of ≥ 2 mm, a value considered clinically critical for preventing colour changes in esthetic restorations and marginal bone loss [4, 44, 45]. These results suggest that i-PRF may effectively support STP in thin phenotypes.

Compared to GT, the effects of i-PRF injections on KTW in natural teeth have been more variable and limited. In short-term studies of 3 months, Tiwari et al. [20] reported a significant increase in KTW from 4.53 to 4.69, Fotani et al. [23] from 5.25 to 5.42, and another study reported a significant increase from 2.72 to 3.14 [21]. Similarly, in a study comparing HA and i-PRF, an increase in KTW was found in both groups [14] However, when the follow-up period was extended to 6 months, two studies reported that i-PRF alone did not produce a significant change in KTW, but significant increases were observed in groups that combined i-PRF with MN [13, 24].One study reported no statistically significant difference in KTW between i-PRF injection and MN + i-PRF at six months [15]. Similarly, Manasa et al. [19] observed no significant difference in KTW between the i-PRF group and the control group. Afshari et al. [48], and Ahila et al. [49] also observed KTW gains during papilla reconstruction using i-PRF. A recent meta-analysis emphasized that injection frequency and interval significantly influence outcomes—for instance, four injections at 10-day intervals yielded improvements, whereas three injections at 7-day intervals did not [36]. In our study, peri-implant thin and thick phenotypes did not show a significant increase in KMW compared to baseline values ​​at 12 months of follow-up. These heterogeneous outcomes may be attributable to inherent biological differences between dental implants and natural teeth, inconsistencies in application protocols, and constraints related to limited sample sizes.

Injectable PRF administered via a semi-surgical approach has been shown to resolve gingival recession in thin phenotypes, achieving complete root coverage in 60% of cases [42]. In contrast, existing gingival recession does not cover but remains stable with minimal invasive i-PRF injection [13]. In our study, REC-H and REC-W values did not show significant changes in either the thin or the thick phenotypes. This result suggests that i-PRF may have a limited effect on soft tissue recession around the implant.

A systematic review and meta-analysis reported that, after follow-up, the mean PES parameters and PES were not significantly different between thin (< 2.0 mm) and thick (≥ 2.0 mm) soft tissues or phenotypes [44]. In another study, PES was associated with patients’ peri-implant soft tissue responses [50]. Happe et al. [51] evaluated PES in the groups receiving autogenous connective tissue grafts and acellular dermal matrix with bovine bone mineral at 12 months, with values ​​of 11.4 ± 1.4 and 10.7 ± 1.5, respectively, without statistically significant differences. When the effect of implant placement after single-tooth extraction in the esthetic zone on PES was evaluated over time, a slight decrease in PES was observed in patients with major alveolar process remodeling and advanced midface recession at 3 months. Connective tissue graft applied during this period contributed to a steady increase in PES in the following months, with significant improvement after the third month [52]. A randomized clinical trial evaluated the effect of connective tissue grafting at the time of immediate implant placement in the esthetic zone and demonstrated that the grafted group had more stable soft tissues and a higher PES value (8 vs. 6.65) at 2-year follow-up [53]. To our knowledge, no studies have evaluated PES outcomes after i-PRF injection. In our study, i-PRF injection increased soft tissue contour, soft tissue colour, and total PES values from baseline to 12 months in all implants. Additionally, while the change in total PES score did not affect the changes in KMW, MT, REC-H, and REC-W parameters between baseline and 12 months, the effect of KMW values ​​at 12 months of follow-up on baseline and 12-month PES-alveolar bone deficiency was significant. This suggests that local soft tissue behavior may have a selective effect on esthetic outcomes. However, the limited sample size limits the generalizability of this relationship and suggests that correlations between some parameters may not have been revealed.

The current literature on i-PRF shows significant methodological variability regarding injection sites, measurement points, follow-up durations, and patient selection criteria. For example, GT measurements have been taken at different locations, such as 1.5 mm from the gingival margin [18] or at multiple points [19], while some studies did not account for tooth position [13, 15]. Additionally, factors like age, systemic disease, and smoking have been identified as variables influencing outcomes [19]. In our study, measurements were made using the midpoint between the free gingival margin and the mucogingival junction as the reference point, and clinical variables such as implant location, systemic conditions, and smoking habits were considered. Although the small sample size limits generalizability, the 12-month follow-up offers valuable long-term data on i-PRF application around implants, addressing a notable gap in existing short-term studies.

Several limitations should be considered when interpreting the results of this study. After the explanation of the study’s aim and procedures, patients who met the selection criteria but declined to participate were excluded. The reduction in sample size from the originally planned 46 implants to 36 during follow-up, as well as the numerical imbalance between groups (25 vs. 11), represent notable limitations. This reduction primarily resulted from the application of strict eligibility criteria intended to maximize data reliability and standardization. Patients who failed to fully comply with the study protocol or were unable to attend follow-up visits due to logistical issues (e.g., transportation difficulties or scheduling conflicts) were excluded, thereby increasing methodological rigor while reducing the sample size. It is important to note that such logistical challenges and follow-up losses (i.e., attrition bias) are common in clinical research. To further enhance the reliability of these findings, GEE analysis were employed, as this method is specifically robust for handling longitudinal data with unequal group sizes. Nevertheless, these findings should be interpreted as a preliminary analysis, and future large-scale prospective studies are warranted to increase the generalizability of the findings and further validate these associations. Furthermore, it is important to support this technique with controlled clinical studies to more comprehensively evaluate its effectiveness on the soft tissues surrounding the implant. Patient-reported experience measures and patient-reported outcome measures were not evaluated in our study; future studies that include these measures will be valuable for assessing treatment success from the patient’s perspective. Longer follow-up periods are needed for the long-term evaluation of keratinized mucosa width and peri-implant soft tissue stability. Moreover, while VSDs were assessed at baseline, they could not be repeated at 12 months, limiting the interpretation of changes over time. Finally, studies investigating the effects of variables such as injection site, application frequency, and i-PRF application time intervals on efficacy will also make important contributions to the field. It is recommended that larger sample sizes are used, that the follow-up period is extended, and that histological or biochemical verification is used to further researchs.

## Conclusion

In this study, different results were obtained for soft tissue phenotype modification with multiple i-PRF injections between thin and thick phenotypes. An increase in the mucosal thickness parameter was observed only in the thin phenotype group, while the MT and KMW values remained stable in the thick phenotype group. When all implants were evaluated, it was concluded that both PES and soft tissue parameters improved from baseline to 12 months.

According to the preliminary results of this study, i-PRF injections can be used as a potential supportive non-invasive method to optimize implant site tissue quality and aesthetic results, especially in thin phenotype cases. However, more studies with controlled groups and larger sample sizes are needed to support the long-term efficacy and biological stability of these effects.

## Data Availability

The datasets generated during and/or analysed during the current study are available from the corresponding author on reasonable.
